# Resituating the ethical gaze: government morality and the local worlds of impoverished Indigenous women

**DOI:** 10.3402/ijch.v72i0.21207

**Published:** 2013-08-05

**Authors:** Caroline L. Tait

**Affiliations:** Department of Psychiatry, University of Saskatchewan, Saskatoon, Canada

**Keywords:** ethical policy, First Nations, child welfare, addictions, mental illness, government policy

## Abstract

**Background:**

Over generations, government policies have impacted upon the lives of Indigenous peoples of Canada in unique and often devastating ways. In this context, Indigenous women who struggle with poverty, mental illness, trauma and substance abuse are among the most vulnerable, as are Indigenous children involved in child welfare systems.

**Objective:**

By examining the life history of Wanda, a First Nations woman, this article examines the intergenerational role that government policies play in the lives of impoverished Indigenous women and their families. Questions of moral governance and responsibility and the need for ethical policies are raised.

**Design:**

The life narrative presented in this article is part of a larger qualitative research programme that has collected over 100 life histories of Indigenous women with addictions and who have involvement with the child welfare system, as children or adults. Wanda's life story exemplifies the impact of government policies that is characteristic of vulnerable Indigenous women and draws attention to the lack of ethical standards in government policymaking in child welfare, public health and mental health/addictions.

**Results:**

The path to recovery for Canadian Indigenous women in need of treatment for co-occurring mental disorders and substance addiction is too frequently characterized by an inadequate and ever shifting continuum of care. For those who feel intimidated, suspicious or have simply given up on seeking supports, a profound invisibility or forgetting of their struggle exists in areas of government policy and programming provision. Living outside the scope of mental health and addiction priorities, they become visible to the human service sector only if they become pregnant, their parenting draws the attention of child and family services (CFS), they need emergency health care, or are in trouble with the law. The intergenerational cycle of substance abuse, mental illness and poverty is commonly associated with child welfare involvement, specifically practices that place the health and well-being of Indigenous children at risk. In order to break this cycle, close attention to implementation of ethically based policies and best practice interventions is required.

**Conclusions:**

From an ethical policy perspective, the focus of government policies and the practices they generate must be first and foremost to ensure that individuals, families and groups are not left worse off than prior to a government policy impacting upon their life. Furthermore, the impact of living a life determined by multiple government policies should not be a story of individual and family devastation, and government policies should not be the most significant determinant of health for any group of people.

The path to recovery for Canadian Indigenous women in need of treatment for co-occurring mental disorders and substance addiction is too frequently characterized by an inadequate and ever shifting continuum of care (1,2). For those who feel intimidated, suspicious or have simply given up on seeking supports, a profound invisibility or forgetting of their struggle exists in areas of government policy and programming provision (3–5). Living outside the scope of mental health and addiction priorities, they become visible to the human service sector only if they become pregnant, their parenting draws the attention of child and family services (CFS), they need emergency health care, or are in trouble with the law (5,6).

Throughout Canada, elevated morbidity and mortality rates among impoverished Indigenous women are unacceptably high (7,8). For decades, rates of substance abuse and mental illness among this subgroup have constituted a population-based mental health crisis. However, despite a substantial body of research illustrating the enormous human, social and economic cost associated with the crisis (1,5,9,10), comprehensive women-centred and culturally safe interventions are limited and in some regions non-existent.

Anthropologist Dara Culhane argues that a “neo-liberal mode of governance selectively marginalizes and/or erases entire categories of people through strategies of representation” (1, p. 595). For example, metaphors of “investing,” “high-performance,” “accountability” and the adopting of “quality improvement” approaches developed in the manufacturing sector dominates contemporary approaches to health care and social welfare reform (11,12). However, emphasizing system “efficiencies” while operating within 4-year political cycles, challenge managers to implement comprehensive “evidence-based” interventions that require longer term applications and the support of multiple government ministries. Coordination between government ministries responsible for the human service sector is near impossible within the existing bureaucracies and represents a significant stumbling block to addressing poverty and other complex social problems. Added to this are jurisdictional lines that divide up governments’ responsibility for Indigenous peoples along federal and provincial/territorial lines, or across government ministries. Creative and innovative “evidence-based” initiatives become mired in bureaucratic inertia, jurisdictional disputes and short-term political mandates. As a result, the application of “best practices” and policy recommendations are more often watered down to actions that support the limitations of the bureaucracy rather than implemented to evidence-based standards (2,13).

In the current neo-liberal context, individuals labelled as living “high risk” or “unproductive” lifestyles are viewed as poor investments with limited positive return to the broader society. However, the needs of this group are complex and require intense (often costly and long-term) interventions to improve their lives (14,15). Keeping individuals on the margins of care through policies that create, rather than dismantle, barriers and gaps to access support services not only saves on immediate (but not necessarily lifetime) health care and social welfare spending, it also sends a message that the government's primary concern is with the “worthy” and not the “undeserving” or “non-contributing.” This message has strong societal support in a country where prevailing attitudes see impoverished Indigenous women as “liabilities” (e.g. they give birth to too many babies that they cannot care for; are habitual welfare recipients) and never as “assets” (e.g. educated, employed taxpayers) to Canadian society.

This article reconsiders the complexities that shape the lived experiences of Indigenous women who simultaneously struggle with poverty, violence, trauma and addictions. The life narrative of Wanda, a First Nations woman, captures the intersecting factors that shape the lives of women who are essentially invisible and unwanted within Canadian society. Wanda's life has been shaped by government policies: from residential school policies that debilitated her parents; to CFS policies that permitted her to be moved from foster home to foster home while failing to safeguard against abuse; to policies that funded short- but not long-term residential treatment for her addiction.

As Wanda's life story reveals, governments intervened in her troubled life at times of “crisis” and in doing so very narrowly defined risk, the ethical management of it, and excluded other mitigating factors. The enormous personal toll for Wanda was lost in technocratic government responses (or non-responses) that were marked by institutionalized race, sex, and class discrimination, and by her own devalued self-worth and diminished moral right to societal support. Drawing attention to societal inequities, Wanda's narrative raises questions of moral governance and responsibility: “Are federal and provincial governments responsible for the life of pain and suffering experienced by Wanda, if she is not?”; “Where are the ethical checks and balances within government policies that take seriously beneficence and non-maleficence for all people?”; and, “If the government failed to safeguard against life-long harm to Wanda when she was a foster child, does it not have a moral obligation to ensure that as an adult she has enhanced supports to address the trauma she endured while in the State's care?”

## Wanda

When I met Wanda she was living in a poverty-stricken neighbourhood in Saskatoon.^1^ She was among a subgroup of women described by outreach workers as “falling through the cracks” of health and welfare systems. At the age of 34, Wanda was confined to a wheelchair after being hit by a car. Her health was rapidly deteriorating and she was living with severe chronic pain. While physiotherapy initially helped, she stopped attending 5 months into her treatment because she felt judged by the therapists about her substance abuse.

Wanda's life is full of multiple challenges, including getting around in her non-electric wheelchair, living on a small disability allowance, and staying safe on the streets. She states: *It's a battle. I try to stop [using substances] but I sit down sometimes and everything just builds up inside me. All my problems are coming and I just do that to forget*.

As a young child Wanda lived on a northern reserve. Both her parents had attended residential schools, returning to their reserve as young troubled teenagers. Wanda remembers never having food in their house, her parents always drinking and the children often being left alone. When she was 4, CFS apprehended Wanda and her siblings, placing them with different families. In foster care, Wanda experienced multiple placements in homes run by non-Indigenous foster parents. While most of the homes were supportive, in 2 of them she was repeatedly sexually and physically abused by the foster father and in both cases the abuse went undetected by CFS. The frequent moves of homes and schools left Wanda feeling isolated and alone, unable to fight back against the abuse.

Wanda was 14 when she started abusing alcohol with other foster children. She used throughout her adolescence and into adulthood, with periodic breaks. While it must have been obvious to some of the adults in her life that she was struggling, Wanda was never offered therapeutic or other supports by CFS. Wanda “aged out” of foster care with a growing substance abuse problem, untreated psychological trauma, and no connection to her biological family, her reserve, or to any of her foster families.

At the age of 18, Wanda became pregnant. She was young, alone, a street worker, and getting high every day. As with many pregnant girls in similar situations, Wanda avoided prenatal care, fearing judgement because of her lifestyle. With no support from the father of her baby, Wanda spent the first months of her pregnancy drinking, drugging and “working the streets.” Fortunately, a social worker became aware of her pregnancy and helped her to enter residential addiction treatment. While the treatment centre gave Wanda stability, it was a 30-day programme with limited aftercare or transitional supports. Once she left the treatment centre, Wanda quickly fell back into the same lifestyle and started using again. The gains that she made in treatment were quickly lost and her relapse fuelled feelings of low self-worth, guilt and shame.

With the birth of her daughter, Wanda tried to parent, however she struggled as a young mother with no supports. CFS eventually stepped in and apprehended her daughter triggering an escalation of Wanda's substance abuse (no supports for her grief and loss) and reliance on prostitution (reduction of social welfare benefits). Within a short period, Wanda's daughter was placed for adoption.

After a few hard years, Wanda married and moved to Edmonton with her husband. This was a relatively stable time even though they struggled financially and were both using heavily. They had 2 daughters and Wanda attended an outreach programme during both pregnancies where she felt genuinely supported. Shortly after the birth of their second child, Wanda separated from her husband because of his infidelity. After her separation, she managed to parent her children and keep her substance abuse under control. However, her substance abuse escalated again when she began a new relationship:

Well I did [quit using substances] when my girls were small and living with me. Well, I did it with them [used substances when pregnant] but then after that I didn't do it for about two or three years. And then I was living with this abusive man. He beat me up all the time and I started doing everything again. Then I phoned my sister and she came and got me. I came to Saskatoon and I started using and I lost them [her daughters to CFS].

The move to Saskatoon, while helping Wanda to get away from an abusive relationship, marginalized her further. She began working the streets and her substance abuse escalated. CFS apprehended Wanda's daughters and after she failed to complete addiction treatment, they moved the girls to Edmonton to live with their father. Alone and without her children, she moved into a run-down rooming house, increased her substance use and worked the streets until the birth of her son. While different supports were offered to her, including referral to addiction treatment and prenatal care, Wanda's depression was so severe that it prevented her from pursuing these supports.

At the age of 30, Wanda consented to a tubal ligation immediately following the birth of her son. The nurses told her that her baby was born with debilitating health problems resulting from her substance abuse. Out of guilt and shame Wanda consented to a tubal ligation, her son was taken by CFS, and she left the hospital without a referral to either addiction treatment or grief counselling:

I got my tubes tied ‘cause I didn't want to put more pain on my kids. I really punished my boy a lot. I told them to do it. When I seen what I did to my boy, I said, “Tie my tubes ‘cause I caused him lots of pain,” … they didn't even let me hold him … they just took him away …. I went to court but I didn't get him back. He can think, but he's got to be on medication ‘cause I did lots with him … with my boy I had no support ‘cause I left my husband. I was pregnant with him, I was working on the street with him. I was using drugs.

Once her son was apprehended and the tubal ligation completed, Wanda's needs became a low priority within the service milieu. It was only after she was hit by a car years later that she received some attention. However, the support given was mainly in the form of physiotherapy rather than a holistic approach that addressed her overall needs. When Wanda stopped going to her physiotherapy, she lost all contact with the health care system and the only support she has in her life is an under-resourced outreach programme with limited hours of operation and no wheelchair accessibility.

The only real happiness that Wanda can recall is when she was with her children. However, her narrative of being a mother is marked by guilt, shame and regret. Over the years, Wanda attended multiple addiction treatment programmes, all in the hope of either keeping or regaining custody of her children. Once the children were permanently removed from her care and she was no longer able to get pregnant, Wanda stopped trying to overcome her addiction. No longer at risk of giving birth, the system lost interest in Wanda's recovery and strategies to motivate her to address her addiction all but disappeared.

## Invisibility

Wanda is one of a number of “invisible” Indigenous women who live in Canada's most impoverished and violent neighbourhoods. Despite battling with addictions and mental illness, they face multiple barriers and service gaps when seeking supports (10,16,17). Post-treatment care is also limited and commonly the resources available are under-funded and lack women-specific programming (17–19). Women like Wanda almost never meet the policy criteria for other supportive services simply because they are neither pregnant (and cannot become pregnant) and do not have children in their care: 2 criteria which are required for entrance into almost all government-funded outreach programming for women (20–22).

Despite their invisibility this is a vulnerable and high-service need population, who without supportive interventions, inevitably experience declining health and risk of premature death (10,15,23–25). Co-occurring health problems noted in addicted women include, cirrhosis, psychiatric hospitalizations, anaemia and poor nutrition, tremors, gastrointestinal bleeding, alcohol-related cancer, hypertension, obstructive pulmonary disease, alcohol/drug-related cognitive deficits and HIV (2,26). Lifetime co-occurrence of mental illness is high and often occurs prior to the onset of substance abuse with childhood abuse being a primary precipitating factor (5,27–29). Despite the high needs of this population, in many settings Indigenous women who abuse substances are considered social “throw aways.” As they move through life, they are increasingly stigmatized and marginalized, making it difficult for them to seek out and sustain treatment regimes (1,5,30). Their diminished social position further places them at elevated risk for violence and exploitation (2,3).

For women living in northern and remote communities, they live in a service environment that is most often lacking in the range of supports available in the south (e.g. residential addiction treatment, detox, mental health outreach, transitional housing and shelters). If Wanda wants to return to her northern reserve, it is likely there would be limited support for her mental illness, addiction or the injuries from the car accident. The ties with her community have also been severed years ago, requiring Wanda to prove her citizenship and negotiate re-entry. At this point in her life, it is unlikely that Wanada will try to move “home” and more likely she will remain socially isolated, without family or community.

## Living policy

In discussing the links between past government policies and the current state of mental health among Canadian Indigenous peoples, colleagues and I wrote in 2000: “Some of these policies were well intentioned, but most were motivated by a condescending, paternalistic attitude that failed to recognize either the autonomy of Aboriginal peoples or the richness and resources of their cultures. The cumulative effect of these policies has, in many cases, amounted to near cultural genocide. The collective trauma, loss, and grief caused by these short-sighted policies are reflected in the endemic mental health problems of many Aboriginal communities and populations across Canada” (31, p. 609). Despite national consensus that government policies have had devastating effects on Indigenous peoples (5, p. xv), our country struggles with sincere acknowledgement and reconciliation. For example, more Indigenous children are wards of the state today than was the case at the height of the residential school era (32,33). This is occurring despite damning research evidence that directly correlates foster placement with elevated risk of behavioral and social problems in adulthood (34–36). While it is well acknowledged that CFS systems perpetuate their own brand of harm upon Indigenous children (33,37,38), there is an absence of societal moral outcry despite frequent media reporting on the problems with CFS. As a result, governments lack motivation to undertake meaningful change, including implementing ethical policies in CFS to bolster prevention and reduce childhood trauma brought on by apprehension procedures, multiple foster placements and foster home overcrowding. In areas of mental health and addictions, ethical policies could include reducing barriers to therapeutic care for all individuals, increasing the abilities of outreach programmes to support high-risk clients in treatment, the provision of intensive long-term aftercare that includes safe housing; and, reunification of children with their parents even when a parent is not in a position to be the primary guardian of that child. Attempts by Indigenous leaders and advocates to make governments more accountable and ethical in areas of health and CFS have been met by government stonewalling, and in some instances lengthy legal battles (38).

It is easy to look at the lives of women like Wanda and see the myriad of government policies across generations that directly and indirectly contribute to ill health, social despair and life-long inequities. If this is the case, then how can we be assured that present-day policies are not equally as damaging? For example, it is not uncommon for children who linger in foster care to experience multiple foster placements or moves between their biological home and foster care (33,34). In some cases, young adults estimate that they were moved upwards of 20–30 different times as children in care, begging the question as to who is really looking out for the best interest of these children both now, and when they reach adolescence and adulthood (39)? Policies that allow multiple foster placements to go unmonitored ignore compelling medical evidence documenting the elevated health and social risks caused to the child by this practice (32–34). The policy and the resulting practice support the needs of the bureaucracy far more than it supports the needs of the child, and when used in the extreme, can result in life-long negative consequences.

In a country as historically wealthy and prosperous as Canada, how is it that certain families are condemned at the hands of their own governments’ policies, to live the life that Wanda and her family have endured? Over 4 generations, Wanda's family's destiny has largely been determined by government policies and interventions despite their resilience and attempts to resist the assaults. From an ethical policy perspective, the focus of policy and the practices they generate must first and foremost ensure that individuals, families and groups are not left worse off than before government policies impact upon their lives. Furthermore, the impact of living a life determined by multiple government policies should not be a story of individual and family devastation, specifically when presumably the state is acting in the “best interest of the child” in cases of CFS protection, or providing “best practice” care to help individuals recover from mental illness and addictions.

As a basis for understanding Wanda's life, this article asks, “Have any of the lessons from the failure of past government policies yielded more humane and ethical government policies and interventions when engaging vulnerable Indigenous peoples?”. Wanda's story unfortunately suggests not; at least not in ways that are meaningful and sustainable to her and her family, or in ways that respect the abilities of Indigenous leaders and front-line workers to know best how to improve the lives of their people.

## Conclusion

The circumstances of impoverished Indigenous peoples are intimately interwoven from birth with historical and contemporary government policies and the resulting consequences of poverty, addiction, trauma, mental illness and entrenched structural violence. In today's political climate, positive reforms to government policies and programming are limited and social welfare benefits have not kept up with inflation, forcing more people into high-risk lifestyles with no meaningful way out of poverty. In remote and northern communities, the lack of voluntary sector supports such as food banks, shelters and transitional housing adds to the vulnerability of the poor and marginalized.

Women with chronic substance abuse and mental illness are at high risk of dying prematurely (10,40,41). Their health and social needs reach far beyond basic outreach services and unless forms of intensive, culturally safe therapy is offered in a place where they feel safe to begin to rebuild (or build for the first time) their lives, and unless this support is provided for extended periods of time (possibly the rest of their life), it is unlikely that their lives will improve. The question for Canadian society is therefore, not one of “efficiencies,” “quality improvement” and “accountability,” rather, when we consider the lives of vulnerable peoples in our society we must ask, “What are our moral values as Canadians?”, “Are we as a society, morally driven to prioritize our most vulnerable citizens; those most difficult to help, and who require our commitment of both time and resources?”, “Do we expect our governments to work towards beneficence and away from maleficence in each and every policy that targets our most vulnerable?”.

Unfortunately, even with a commitment to ethically driven policies and services there are no guarantees that individuals will fare better if they remain in neighbourhoods and communities where poverty is endemic and only limited options exist for them to transform their lives. The following words of Nelson Mandela pushes us one step further, “Like slavery and apartheid, poverty is not natural. It is man-made and it can be overcome and eradicated by the actions of human beings. And overcoming poverty is not a gesture of charity. It is an act of justice. It is the protection of a fundamental human right, the right to dignity and a decent life. While poverty persists, there is no true freedom” (42).

## Footnotes

1 To protect Wanda's confidentiality, the name used is a pseudonym, as are the locations. Wanda's life narrative is from a larger study and exemplifies the life narratives that I have collected over the past 18 years from Indigenous women who have lost their children to CFS.
